# Influence of Two Various Durations of Resistance Exercise on Oxidative Stress in the Male Rat’s Hearts

**DOI:** 10.15171/jcvtr.2015.32

**Published:** 2015-11-28

**Authors:** Rafigheh Ghiasi, Mustafa Mohammadi, Javad Ashrafi Helan, Seied Raziallah Jafari Jozani, Shima Mohammadi, Akbar Ghiasi, Roya Naderi

**Affiliations:** ^1^ Drug Applied Research Center, Tabriz University of Medical Sciences, Tabriz, Iran; ^2^ Department of Pathobiology, Faculty of Veterinary Medicine, University of Tabriz, Tabriz, Iran; ^3^ Student of Pharmacy, Pharmacy School, Tabriz University of Medical Sciences, Tabriz, Iran; ^4^ Faculty of Management, Zabol University of Medical Sciences, Zabol, Iran; ^5^ Department of Physiology, Tabriz University of Medical Sciences, Tabriz, Iran

**Keywords:** Resistance Exercise, Oxidative Stress, Duration of Exercise, Rat

## Abstract

***Introduction:*** The previous studies have suggested that alteration in oxidative stress and antioxidant defense depends on various factors, such as mode, intensity, frequency and duration of exercise. In this study, we compared the effects of two various durations of resistance exercise (1 month and 4 month) on oxidative stress and antioxidant status in cardiac tissue.

***Methods:*** Thirty Wistar male rats divided into 3 groups: control (sedentary), exercise-1 (regular exercise for 1 month) and exercise-2 group (regular exercise for 4 months). After the final to the experiment, the rats were anesthetized, and then blood and heart samples were obtained and used to determine glutathione peroxidase (GPX), superoxide dismutase (SOD), malondialdehyde (MDA) and biochemical estimation.

***Results:*** MDA levels between control and exercise-2 groups showed no significant difference, hence, MDA level in exercise-1 group was higher compared to control group (*P* < .01). The heart GPX activity increased significantly in exercise-2 group regarding other groups (*P* < .01). The SOD activities of groups were similar. Creatine kinase (CK) and lactate dehydrogenase (LDH) concentrations increased in the exercise-1 compared to the other groups (*P* < .01).

***Conclusion:*** Our results indicate that in heart, the adaptation and alteration in oxidative stress and cell injury level depend on duration of exercise.

## Introduction

Reactive species (ROS) produce along with body metabolism and have essential role in many cellular processes, like signaling, detoxification of xenobiotic, apoptosis and provoking of antioxidant activity and repair processes.^1,2^ Oxidative stress is described as an inequality between free radical assembly and the antioxidant enzymes production in body that leads to cell injury.^3^ Conversely, ROS are also believed to be complicated in a number of pathological processes like cachexia, atherosclerosis, cancer, ischemia-reperfusion, and inflammatory neurodegenerative diseases.^2,3^ Many studies indicated that exercise training is beneficial to conserve physical well-being. It is now clear that people who do regular exercises, are less threatened by cardiovascular diseases, and in comparison with sedentary individuals, they benefit from better quality of life.^3^ Nevertheless, increased cellular metabolism during exercise results in ROS creation and this process can be hazardous since they attack membrane lipids.^4,5^ It was revealed that regular exercise can intensify the production of free radicals and cause oxidative stress.^5^ It has been shown that exercise can increase the activities of antioxidant enzymes and can reduce the damage resultant from free radicals.^5^ The alteration in antioxidant defense depends on variety of factors, like mode, intensity and frequency of exercise, even acute exercise enhances oxidative stress. It has been reported that regular exercise program can increase the activities of antioxidant enzymes.^3,4^ Regarding the fact that regular exercise training is related to numerous health profits, hence it can be regarded as a strong physical stressor tended to augment oxidative cellular injury, likely because of increased production of ROS.^6^ Short time training can cause the activation of several different structures of free radical production and may be severed into both initial (e.g., the leakage of electrons inside the mitochondria during aerobic respiration, amines and prostanoid metabolism and the enzymes including NADPH oxidase or xanthine oxidase), in addition to secondary origins (e.g., phagocytic cells, disturbance of iron have within proteins, and extreme calcium collection).^7^ Short term anaerobic exercise can enhance the level of lipids oxidation and induce oxidative stress.^7,8^ Creatine kinase (CK) and lactate dehydrogenase (LDH) are non-plasma particular enzymes that are liberated into plasma as the result of the desolation of the cell membrane by oxidative stress or tissue injury proceeded by the direct obliteration of the cell membrane and tissue necrosis.^9-11^ Thus, alterations in the activity of CK and LDH are indicatives of membrane injury resulted from oxidative stress or tissue impairment.^9^ Recently, CK and LDH are connected to reactive oxygen species (ROS) due to the fact that such enzymes are activated when cells are impaired by inflammatory conditions and oxidative stress.^9^ Previous studies demonstrated that heart has a lower level antioxidant defense (antioxidant enzymes like superoxide dismutase…), than the other tissues, thus it is vulnerable to be damaged by ROS.^12^ In various tissues, the Long-term exposure to increased oxidative stress leads to adaptation by stimulating the antioxidant enzymes activity such as superoxide dismutase (SOD), glutathione peroxidase (GPX) in exercised rats rather than unexercised rats.^13^ Having to say, the goal of the current time study was to compare the effect of two various duration exercises (1 month and 4 months) on oxidative stress and antioxidant status in blood and in cardiac tissue.

## Materials and Methods

Thirty Wistar male rats (250-300 g) were provided from laboratory animal house at Tabriz University of Medical Sciences. They were kept in animal room at 22ºC–24ºC with food and water provided *ad libitum*. Handlings and caring of animal were matched by rules approved by animal care committee of Tabriz University of Medical Sciences. The rats were divided into 3 groups (n=10): control (sedentary animals age matched with exercise groups), exercise-1 (regular exercise for 1 month) and exercise-2 (regular exercise for 4 months). In exercise groups, animals were exercised as stated by the model designated by Tamaki et al,^14^ with some alterations. Rats were put perpendicularly in a squat-training device cylinder (RatWLI009, Tajhiz Amaya Pooya Co, Iran) so they could be placed upright on their hind limbs in reaction to electrical motivation and lift the piston which was directly positioned over their heads. An electrical stimulus (20 V, 0.3-second duration at 3-second intervals) was used to the rat’s tail through a surface electrode. After 1 week of adaptation, the exercise groups of rats exercised for 4 sets of 12 repetitions per day, with a 90 minutes interval between each set, 5 times per week for 4 and 16 weeks.^15^ Heaviness of rats in trained group measured daily and 120% of its body weight (approximately 70% of the maximum load that the rats were capable to exalt following electrical motivation) was used to conclude the weight of the piston. Piston movements for each rat were recorded by a distance sensor located above the piston and the each rat’s performance was calculated daily by reproducing the piston weight and piston movement. At the end of experiments all animals were anaesthetized with pentobarbital sodium (35 mg/kg intraperitoneal injection),^16,17^ then heart tissue and blood were collected to determine GPX, SOD, malondialdehyde (MDA), LDH and CK.

### Tissue Processing and Homogenate Preparation

Blood samples were obtained from the inferior vena cava and kept in tubes at −70°C to demonstrate blood SOD and GPX activities. Hearts were excised, frozen in liquid nitrogen and stocked at deep freeze (-70°C) for subsequent measurements. For antioxidant activity calculation, heart samples were homogenized in 1.15% KCl solution. Homogenates centrifuged at 1000 rpm for 1 minute at 4°C. The tissue homogenate was then stored at −20°C for GPX, SOD activities and MDA measurements.

### Determination of Antioxidant Enzymes

Whole blood samples were utilized to determine of GPX and SOD. SOD activity was determined using a commercial kit (RANSOD, Randox Co., Antrim, United Kingdom) based on Delmas‑Beauvieux et al^18^ SOD activity was measured at 505 nm by a spectrophotometer (Pharmacia Biotech; England). In such method, xanthine and its oxidase were employed to produce superoxide radicals that would respond with 2‑(4‑iodophenyl)‑3 (4‑nitrophenol) ‑5‑phenyl tetrazolium chloride (ITN) to create a red formazan dye. Concentrations of substratum were 0.05 mmol/L for xanthine and 0.025 mmol/L for ITN. SOD activity was measured by the inhibitory degree of this reaction. After calculating the percentage of inhibition via specific formula, SOD activity value was calculated compared to the standard curve and was expressed as U/g hemoglobin (Hb) in blood and U/mg Pr. in tissue. GPX activity was determined using commercial kit (RANSEL, Randox co., Antrim, United Kingdom) according to the method of Bradford.^18^

GPX catalyzes the glutathione oxidation (at a concentration of 4 mmol/L) by cumenehydro peroxide. In the attendance of glutathione reductase (at a concentration ≥0.5 units/L) and 0.28 mmol/L of NADPH, oxidized glutathione is at once transformed to its reduced form with accompanying oxidation of NADPH to NADP+. The reduction in absorbance at 340 nm (37°C) was weighed by a spectrophotometer (Pharmacia Biotech; England), and then GPX level was calculated by its formula and expressed as U/g Hb in blood and U/mg Pr in tissue.^18^

### Malondialdehyde Assessment

MDA, the final-output of lipid peroxidation, was measured in the blood samples and tissue extracts based on Esterbauer and Cheeseman method, MDA responses to thiobarbituric acid and produces a pink pigment that has a maximum absorption at 532 nm.^19,20^

### Biochemical Measurement

Blood was sampled for LDH and CK measurement. Amounts of LDH and CK were determined by an autoanalyzer Technicon DAX method. The LDH activity definition is established on the assessment of the changing of pyruvate to L-lactate process by observing the oxidation of NADH.^21^ The ratio of oxidation is equivalent to LDH activity. The activity is supervised by weighing the reduction in absorbance at 340 nm. In the determining CK activity the enzyme responds with creatine phosphate and adenosine diphosphate to create adenosine triphosphate that is linked to the hexokinase/guanosine diphosphate reaction producing NADPH.^22^ The CK activity is identical to the ratio of raise in the amount of NADPH. The reaction is supervised at 340 nm. GPX activity was demonstrated using a RANSEL kit (Randox labs Crumlin, UK) according to the Paglia and Valentine method.^23^

### Statistical Analysis

Results were statistically examined by one-way analysis of variance (ANOVA) followed by Tukey test. The significant rank was set at *P *< .05. Data are expressed as mean± SEM.

## Results

**Figure 1:**
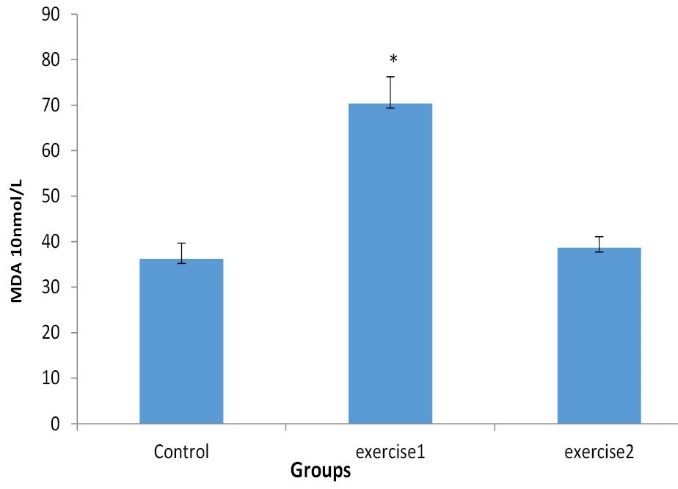
Effect of exercise during 1 and 4 months (exercise-1 and exercise-2 groups) on MDA levels in heart tissues of rats. Data are expressed as Mean ± SEM for each group (n = 10). *P < .01 when compared to control group.

In heart tissue no significant difference was found in MDA levels between control and exercise-2 groups, but MDA level in exercise-1 group increased significantly compared to control group (*P *< .01). Level of MDA in exercise-2 group appears to be significantly lower in comparison to exercise-1 group (*P *< .01; Figure 1).

**Figure 2:**
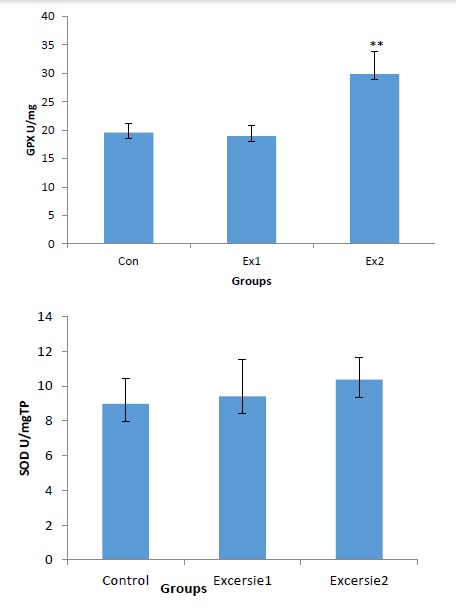
Effect of exercise during 1 and 4 months (exercise-1 and exercise-2 groups) on GPX and SOD levels in heart tissue of rats. Data are expressed as mean ± SEM for each group (n = 7). *P < .01 when compared with control group.

In heart tissue samples GPX activity significantly increased in exercise-2 group compared to the other groups (*P *< .01). GPX activity in exercise-2 group appears to be significantly higher in comparison with exercise-1 group (*P *< .01). In short and long term period, exercise failed to elevate SOD activity significantly. SOD and GPX activities in exercise-1, exercise-2 groups are similar (Figure 2).

Exercise in one month remarkably increased concentrations of diagnostic milestone enzymes (LDH and CKMB) as an indicative for cell damage and tissue injury in comparison with the controls (*P *< .01)

**Figure 3:**
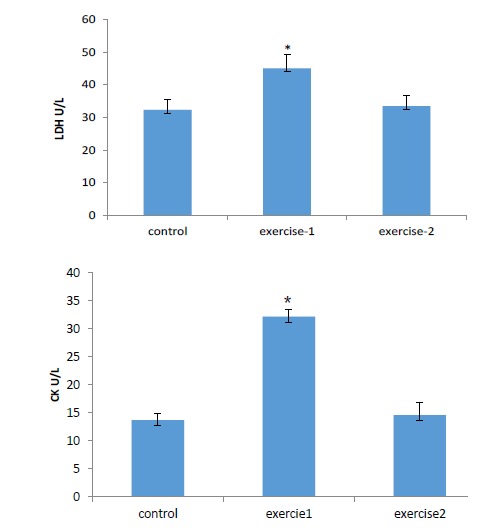
Levels of CK-MB and LDH existed in control, exercise-1and exercise-2 groups of rat’s serum. Data are expressed as Mean ± SEM for each group (n = 10). *P < .01 compared to control group; **P < .05 compared to control group.

Exercise in four month also increased LDH and CK concentrations compared to control but it was not significant. Level of CK in exercise-2 group appears to be significantly lower in comparison to exercise-1 group (*P *< .01; Figure 3).

## Discussion

The present study was planned to evaluate the effect of exercise training that was accomplished at 2 various durations for 1 month and 4 months, and on biomarkers of lipid peroxidation by ROS additional to antioxidant activities and LDH, CK concentrations. The main findings were: Exercise in 1 month increased the MDA, CK and LDH levels. Training at 4 months duration restored antioxidant enzymatic activity (GPX).

MDA is derived from oxidative destruction of lipids in cell membranes, and the alteration in MDA concentration can be an indicator of oxidative cell injury.^24^ It has been reported that the amount of lipid peroxidation depends on the intensity, duration and mode of exercise. For example, a short-term of high-intensity exercise can increase speed of lipid peroxidation which caused by oxidative stress,^25-27^ while long period exercise can diminish lipid peroxidation process and prevents tissue from damaging.^28,29^ The discrepancies between the present findings and those of other results could be related to the duration, intensity and mode of exercise used during this experiment.

GPX protects the cellular and subcellular membranes against the peroxidative damage by elimination of hydrogen and lipid peroxides. In the present study, we detected substantial increase in heart GPX activity of animals trained for 4 months indicating that the tissue antioxidant status was being operated at enhanced level in exercise condition. In consistent with our finding, it was shown that exercise increased the GPX activity in liver, kidney, skeletal muscle and heart.^30^ Increased activity of this enzyme lead to reducing the injuries produced by the enhancement of lipid peroxidation that may act by counteracting the harmful products produced by exercise.^31,32^

It has been shown that exercise can raise blood activities of cytosolic enzymes like LDH and CK^9-11^ due to lack of oxygen or glucose supplying, the cell membrane can be penetrable or may rapture, which cause the enzymes to leak out. Therefore, LDH and CK are utilized as cell injury markers that are liberated into the blood subsequent to oxidative damage of cell membranes or tissue injury throughout extreme training.^10,11,33^ Also it is suggested that the liberation of CK and LDH into the plasma and tissue is the consequence of increased penetrability of the plasma membrane because of lipid peroxidation.^9,34^ Parallel to our results, it has been demonstrated that after exercise, CK blood activity proportional to the level of exercise.^9-11^ Similar to our results, the most noticeable high CK occurs in the less-trained subjects.^10,11^ Enhanced lipid peroxidation seems to be the embarking stage that makes tissue more vulnerable to oxidative injury. This may be account for the discerned membrane damage as confirmed by the high lipid peroxidation. Long-term regular exercise has a preserving effect on the myocardium, diminishing the cardiac damage there by limiting the leakage of these enzymes.

Indeed, short time exercise can produce results different of chronic exercise. Long-term regular exercise-induced adaptations that can produce high levels of antioxidant enzymes, enhanced resistance against oxidative stress, and can reduce cell injury resultant from oxidative stress induced by exercise. We suggest that adaptation and alteration in oxidative stress and cell injury level in heart depend on duration of exercise may be due to reducing of the basic deteriorative reactions produced by lipid peroxidation.

## Acknowledgments

We wish to thank Drug Applied Research Center of Tabriz University of Medical Sciences for it financially support of our study.

## Ethical issues

This protocol was planned based on NIH and Ethics Committee guidelines to the use of animals in research at Tabriz University of Medical Sciences.

## Competing interests

Authors declare no conflict of interest in this study.
